# A framework for plasticity implementation on the SpiNNaker neural architecture

**DOI:** 10.3389/fnins.2014.00429

**Published:** 2015-01-20

**Authors:** Francesco Galluppi, Xavier Lagorce, Evangelos Stromatias, Michael Pfeiffer, Luis A. Plana, Steve B. Furber, Ryad B. Benosman

**Affiliations:** ^1^Equipe de Vision et Calcul Naturel, Vision Institute, Université Pierre et Marie Curie, Unité Mixte de Recherche S968 Inserm, l'Université Pierre et Marie Curie, Centre National de la Recherche Scientifique Unité Mixte de Recherche 7210, Centre Hospitalier National d'Ophtalmologie des quinze-vingtsParis, France; ^2^Advanced Processors Technology Group, School of Computer Science, University of ManchesterManchester, UK; ^3^Institute of Neuroinformatics, University of Zürich and ETH ZürichZürich, Switzerland

**Keywords:** SpiNNaker, learning, plasticity, neuromorphic hardware, STDP, BCM

## Abstract

Many of the precise biological mechanisms of synaptic plasticity remain elusive, but simulations of neural networks have greatly enhanced our understanding of how specific global functions arise from the massively parallel computation of neurons and local Hebbian or spike-timing dependent plasticity rules. For simulating large portions of neural tissue, this has created an increasingly strong need for large scale simulations of plastic neural networks on special purpose hardware platforms, because synaptic transmissions and updates are badly matched to computing style supported by current architectures. Because of the great diversity of biological plasticity phenomena and the corresponding diversity of models, there is a great need for testing various hypotheses about plasticity before committing to one hardware implementation. Here we present a novel framework for investigating different plasticity approaches on the SpiNNaker distributed digital neural simulation platform. The key innovation of the proposed architecture is to exploit the reconfigurability of the ARM processors inside SpiNNaker, dedicating a subset of them exclusively to process synaptic plasticity updates, while the rest perform the usual neural and synaptic simulations. We demonstrate the flexibility of the proposed approach by showing the implementation of a variety of spike- and rate-based learning rules, including standard Spike-Timing dependent plasticity (STDP), voltage-dependent STDP, and the rate-based BCM rule. We analyze their performance and validate them by running classical learning experiments in real time on a 4-chip SpiNNaker board. The result is an efficient, modular, flexible and scalable framework, which provides a valuable tool for the fast and easy exploration of learning models of very different kinds on the parallel and reconfigurable SpiNNaker system.

## 1. Introduction

Learning is crucial for the survival of biological organisms, because it allows the development of new skills, memories, and behaviors, in order to adapt to the information acquired from their local environment. Such high-level changes of behavior are the manifestation of an intricate interplay of synaptic plasticity processes, which lasts from early development throughout the adult life, and is taking place simultaneously and continuously in all parts of the nervous system. Although neuroscience has developed an increasingly better insight into the local plasticity mechanisms at specific types of synapses, we still have a poor understanding of the global effects of plasticity that lead to the emergence of our astonishing cognitive capabilities. Clearly, this is one of the great unsolved questions, not only for neuroscience, but with great implications for fields like philosophy, psychology, medicine, and also for engineering disciplines concerned with the development of artificial intelligent systems that can learn from their environment.

Much of our understanding of the functional effects of local plasticity comes from theoretical and simulation studies of simplified learning rules in neural network models. Most influential is the hypothesis of Hebb (1949), which says that synaptic connections strengthen when two connected neurons have correlated firing activity. This has inspired many classical models for associative memory (Hopfield, 1982), feature extraction (Oja, 1982), or the development of receptive field properties (Bienenstock et al., 1982). Later, the discovery of Spike-timing Dependent Plasticity (STDP) (Markram et al., 1997; Bi and Poo, 1998) has led to a number of models that have exploited the precise timing properties of spiking neurons for receptive field development (Song and Abbott, 2001; Clopath et al., 2010), temporal coding (Gerstner et al., 1996; Guyonneau et al., 2005), rate normalization (Song et al., 2000; Kempter et al., 2001), or reward-modulated learning (Izhikevich, 2007; Legenstein et al., 2008; Friedrich et al., 2011; Potjans et al., 2011). It has also been realized that there is not one standard model for STDP, but that there is a huge diversity of learning rules in nature, depending on species, receptor, and neuron types (Abbott and Nelson, 2000; Kullmann et al., 2012), the presence or absence of neuromodulators (Pawlak et al., 2010; Cassenaer and Laurent, 2012), but also on other factors like post-synaptic membrane potential, position on the dendritic arbor, or synaptic weight (Sjöström et al., 2001).

The discovery that basic effects can be achieved with local learning rules has had a big influence on the development of larger scale learning models that have mapped methods from machine intelligence onto spiking neural networks. Examples include supervised learning methods for classification of visual (e.g., Brader et al., 2007; Beyeler et al., 2013), or auditory stimuli (Sheik et al., 2012), and unsupervised learning methods like Expectation Maximization (Nessler et al., 2013; Kappel et al., 2014), Independent Component Analysis (Savin et al., 2010), or Contrastive Divergence (Neftci et al., 2014). This has opened up the possibility of using spiking neural networks efficiently for machine learning tasks, using learning algorithms that are more biologically plausible than backpropagation-type algorithms typically used for training artificial neural networks.

The increased interest in spiking neural networks for basic research and engineering applications has created a strong interest for larger, yet computationally efficient simulation platforms for trying out new models and algorithms. Being able to easily and efficiently explore the behavior of different learning models is a very desirable characteristic of a such platform. The major problem for computation with spikes is that it is a resource-intensive task, due to the large number of neurons and synapses involved. Synaptic activity, and specifically synaptic plasticity, which might be triggered by every spike event, is dominating the computing costs in neural simulations (Morrison et al., 2005; Brette et al., 2007), partly because the communication and processing of large numbers of small messages (i.e., spikes), is a bad match for current von Neumann architectures. Different strategies to improve the scale and run-time efficiency of neural simulations either rely on supercomputer simulations (Plesser et al., 2007; Wong et al., 2013), parallel general-purpose devices such as GPUs (Fidjeland and Shanahan, 2010) and FPGAs (Neil and Liu, 2014), or special purpose *neuromorphic* hardware (Indiveri et al., 2011). Each solution involves a trade-off between efficiency, reconfigurability, scalability and power consumption.

In this context we present a framework for studying arbitrary plasticity models on a parallel, configurable hardware architecture such as SpiNNaker. The SpiNNaker system (Furber et al., 2006, 2014) has been designed as a massively parallel, highly reconfigurable digital platform consisting of multiple ARM cores, which optimally fits the communication requirements for exploring diverse synaptic plasticity models in large-scale neural simulations. Previous implementations of plasticity on SpiNNaker have been limited in their ability to model arbitrary spike- and rate-based learning rules. Here, we present a new approach for implementing arbitrary plasticity models on SpiNNaker, using a dedicated plasticity core that is separated from other cores that process other neural and synaptic events. Specifically we demonstrate the implementation of three synaptic plasticity rules with very different requirements on the trigger events, and on the need to store or access additional variables for computing the magnitude of updates. We show that the same architecture can implement the rate-based BCM rule (Bienenstock et al., 1982), an implementation of standard STDP based on a model by Morrison et al. (2008), and a voltage-dependent STDP rule suggested by Brader et al. (2007). We compare the efficiency and correctness of the STDP rule to previous implementations on SpiNNaker, and provide the first implementation of BCM and the learning rule of Brader et al. (2007) on this platform. All the experimental results presented in this paper come from implementations of learning rules on a 4-chip SpiNNaker board.

The ability to implement different rules with very different requirements, that are either based purely on spike-timing, on the correlation of firing rates, or on additional voltage signals indicates that the framework can be used as a generic way of implementing plasticity in neural simulations. This new architecture therefore provides an efficient way for exploring new network models that make use of synaptic plasticity, including novel rules and combinations of different plasticity rules, and paves the way toward large-scale real-time learning systems.

This article is organized as follows: the next Section introduces different approaches to model learning, from a theoretical and an implementation point of view. Section 3 describes the SpiNNaker system, the previous solutions for plasticity on SpiNNaker and our novel approach presented in this work. The flexibility of the framework introduced is demonstrated by the implementation of three different rules, presented in Section 4, 5, and 6: Spike-Timing Dependent Plasticity (Gerstner et al., 1996), the rate-based BCM rule (Bienenstock et al., 1982) and the voltage-dependent variation of the STDP rule (Brader et al., 2007). We validate the implementation by replicating classical plasticity experiments, and discuss the performances of each rule in Section 7. The paper is concluded in Section 8, which also provides an outlook toward future applications.

## 2. Learning in spiking platforms

The use of parallelization to mitigate the computational costs and difficulties of modeling large plastic networks has been exploited using different tools and strategies. Using many processors in a supercomputer is an important exploratory solution, which can be used to rapidly implement and test learning rules. However, setting up a Message Passing Interface (MPI) mediating the spike communication is a challenging process on a distributed von-Neumann architecture, because the network infrastructure is optimized for large-frame transfers (Plesser et al., 2007; Wong et al., 2013) as opposed to small spike packets.

Dedicated neuromorphic (Mead, 1989) systems are natural candidates for emulating parallel neural computation. On these systems, circuits modeling neurons and synapses can be replicated using Very Large Scale Integration technology (Indiveri et al., 2011). Synapses usually take up the majority of the resources, in terms of computation and chip area. It is also particularly challenging to design plastic hardware synapses. In the FACETS wafer-scale hardware (Schemmel et al., 2007), for example, the area of plastic synapses is minimized by separating the accumulator circuit for the spike-timing dependency and a global weight-update controller, which drives the update of multiple synapses (Pfeil et al., 2012). Having a separate plasticity engine makes the update slower, but adds flexibility to the plasticity algorithms that can be implemented. The trade-off in this case relates to the controller frequency update, which evolves slower than the neural dynamics, and the precision of the synapses, limited to 4-bits. Despite these limitations the system is capable of modeling a variety of plasticity models, characterized by different weight dependencies. Also, the synaptic resolution is shown to be not-critical in the simulation of a series of network benchmarks. Vogelstein et al. (2002) have introduced a general system where synapses are stored in digital memory with a processor implementing the synaptic update mechanism, while a separate set of ASICs implement the neural integration process. While they demonstrate STDP, more general functions can be implemented using the same scheme.

Brader et al. (2007) proposed a learning rule that captures biological properties such as memory preservation and encoding. Furthermore, it is optimized for efficient implementation in a neuromorphic system. The rule is dependent on the post-synaptic neuron membrane potential and recent spiking activity at the time of a pre-spike arrival. Every synapse has internal dynamics, which drives the weight toward a bistable state. Its advantage for VLSI implementations (Indiveri et al., 2006; Giulioni et al., 2008; Mostafa et al., 2014) lies in its ability to smooth device mismatch by applying a threshold to the internal state variable, in order to set the synapses to one of two possible states. The bistable representation of memory has the additional advantage of being power efficient. The fact that the rule can be computed when a pre-synaptic spike is received reduces the chip area required by a synapse, and consequently increases the number of synapses that can be modeled. This assumes that the synapses are located on the post-synaptic neuron, and have access to the neural and synaptic state variables when a spike is received. This is the case in the VLSI devices mentioned above and also in SpiNNaker (Jin et al., 2010a). A review of different neuromorphic approaches and challenges in designing plastic synapses can be found in Rahimi Azghadi et al. (2014), which discusses power consumption, area requirements, storing techniques, process variation and device mismatch.

Recently, Resistive Random Access Memories, commonly referred as memristors, have raised interest in the neuromorphic community. They are small, power-efficient devices that can be used to store weights and thereby increase the amount of neurons and synapses that can be integrated in a chip. Weight change can be induced by controlling the voltage at the terminals of a memristor, inducing a change in its state and thus modeling a learning rule such as STDP (Zamarreño Ramos et al., 2011) or triplet-based STDP (Mayr et al., 2012). In Indiveri et al. (2013) memristors are used directly to model synaptic dynamics, using them both for computation and memory storage.

There are also difficulties when implementing synaptic plasticity in general purpose hardware. Regarding GPUs Fidjeland and Shanahan (2010), for example, propose a simplified nearest-neighbor pairing scheme with a time-limited STDP window. They continuously accumulate STDP statistics that are then used to update synapses at fixed intervals. In such implementation, increasingly shorter intervals impact performance, lowering the overall spike throughput of the platform. Weight change accumulation is commonly used in other GPU approaches, e.g., in Nageswaran et al. (2007), where the synaptic kernel update is applied every second, and in software simulations (Izhikevich, 2006).

The diversity of approaches for studying synaptic plasticity in hardware, indicates a need for general purpose, massively parallel, and reconfigurable computing platforms. Only this will allow fast prototyping of plasticity rules, and their exploration in large scale models, which can in a second stage directly lead to dedicated hardware implementations.

## 3. A novel framework for plasticity implementation on SpiNNaker

SpiNNaker (Furber and Temple, 2008; Furber et al., 2014) is a digital multi-core, multi-chip architecture designed for the simulation of large neural networks in real time. Each SpiNNaker chip is equipped with a 1Gbit SDRAM and 18 programmable ARM968 cores embedded in a congurable packet-switched asynchronous fabric (Plana et al., 2007).

The SpiNNaker network infrastructure is designed with spiking communication in mind: every chip contains an on-chip Multicast (MC) router capable of handling very efficiently one-to-many communication of spikes (MC packets). The router links every chip to its six neighbours. Each core has a small local tightly-coupled memory (32 kByte instruction and 64 kByte data, ITCM and DTCM respectively). The massive synaptic data required for neural simulations is stored in the shared, off-die SDRAM 128 MByte chip that can be accessed by the cores through DMA requests, for an aggregate read/write bandwidth of 900 MBytes/s (Painkras et al., 2013). The system is designed to scale up to 60,000 chips for a total of over one million ARM cores. The goal of the system is to simulate 1% of the human brain in real time.

**Figure 1:**
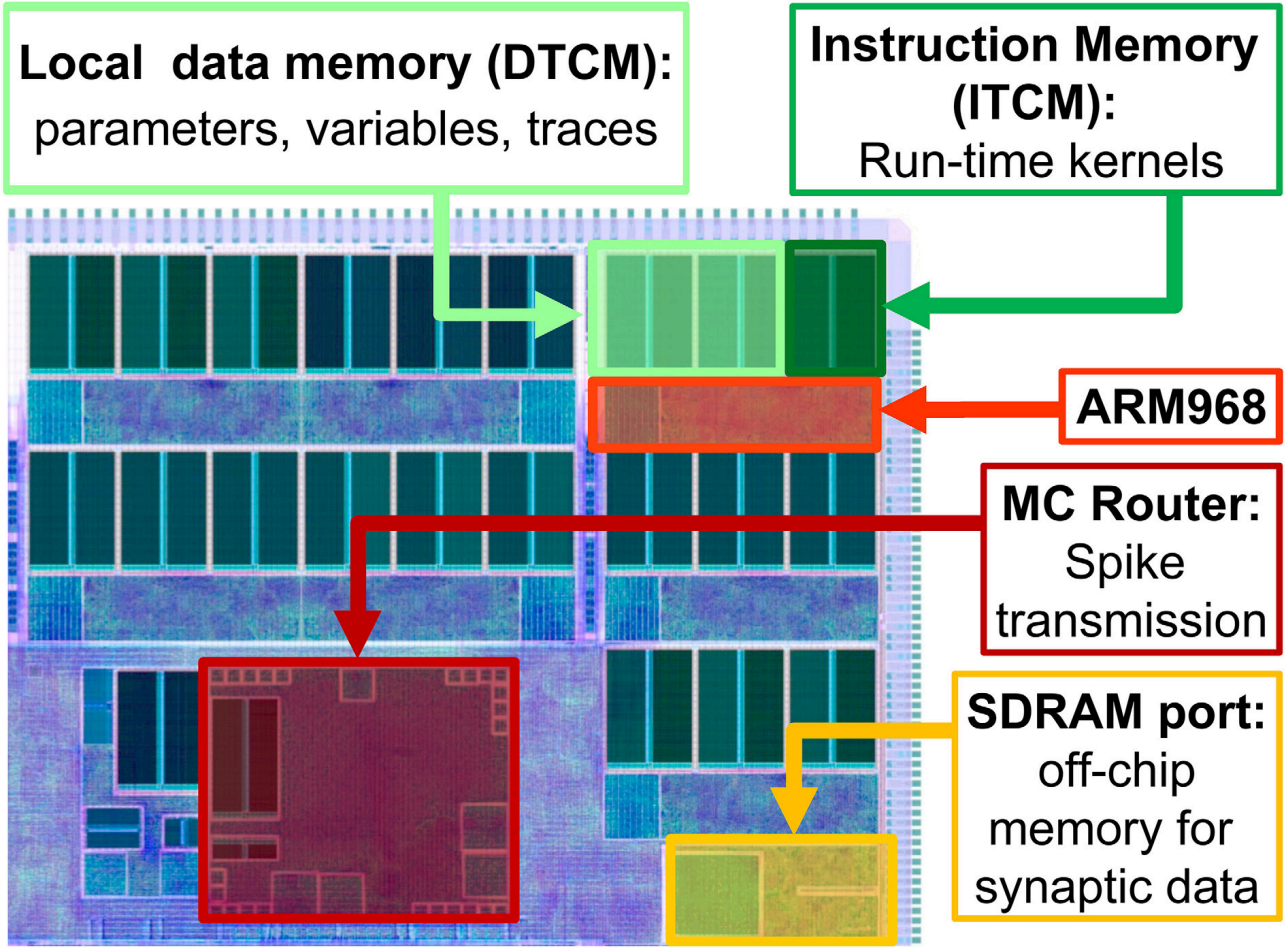
**High-level view of the SpiNNaker chip, showing: the ARM cores with their Instruction and Data Tightly coupled memory (DTCM and ITCM, 32 and 64 Kbyte respectively) to run applications and locally store data; the Multicast (MC) router responsible of spike transmission; the port to the 128 Mbyte SDRAM off-chip memory, containing the synaptic data**.

A high level view of the main chip components is presented in Figure 1. When simulating neural networks, spikes are delivered and processed by the ARM cores, which update the states of the neurons and synapses. A C-based API is used to program neural kernels (Sharp et al., 2011). The API offers an accessible interface to the hardware substrate and to real-time event scheduling facilities, and can be used to write applications that are executed in parallel on the machine. The API promotes an event-driven programming model: the neural kernels are loaded into the ARM cores and are used to configure *callbacks* that respond to *events*. A timer event allows the periodic execution of functions, such as neuron state update. A packet event signals the arrival of an MC packet (spike) and can be used to initiate a request to transfer synaptic data from SDRAM. Finally, a memory event indicates that the requested data is available for processing. The neural kernels are parameterizable and can support different classes of neural models and connectivity patterns. Model specification, system mapping and run-time control is obtained through the PArtition and Configuration MANager (PACMAN, Galluppi et al., 2012), which offers interfaces with two languages extensively used in the neural modeling community: PyNN (Davison et al., 2008), a simulator-independent specification language, and Nengo (Stewart et al., 2009), the simulation tool implementing the principles of the Neural Engineering Framework.

**Figure 2:**
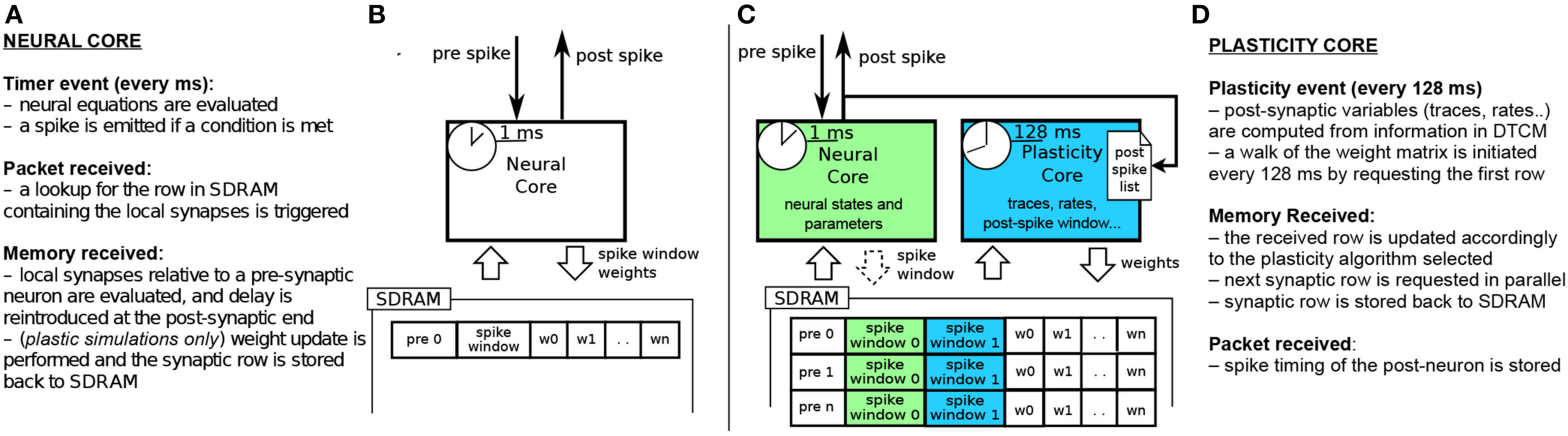
**(A,B)** current STDP implementation on SpiNNaker, following the Deferred Event Driven model **(C,D)** the proposed novel implementation framework for plasticity implementation.

Figures 2A,B shows the current implementation of a neural kernel, highlighting the processes involved: every millisecond, a *timer event* triggers the evaluation of the neural dynamics. A spike is then emitted if a configurable threshold of the membrane potential has been reached. Spikes travel as MC packets through routers on the interconnection fabric and are delivered to the destination cores, triggering a *packet event*. Whenever a packet is received, a memory look-up is initiated to retrieve the relevant synaptic information (such as weight, delay, destination neurons on the core, and type of synapse) from SDRAM, where the connectivity matrix, indexed by pre-synaptic neuron, is stored. When the requested data arrives this creates a *memory event*, and the spike is processed by every post-synaptic core. Due to the limited memory available in the ARM cores, the synaptic weights are only locally available to the core right after a memory transfer from DMA has occurred as a consequence of the arrival of a spike. Therefore, the time available for the weight update process is very short; moreover, since delays are reintroduced at the post-synaptic end, the update process relies on information which might concern the future state of the neuron. This has limited the flexibility of previous approaches for implementing plasticity on SpiNNaker.

### 3.1. The deferred event driven model

The STDP algorithm requires computation whenever a pre spike is received or a post spike is emitted. This causes two relevant issues for the cores running neural simulation on SpiNNaker:

1. Weights are only available in local memory upon the reception of a MC packet signaling that a spike has occurred in one of the pre-synaptic neurons. At the time of a post-synaptic spike such information is stored in SDRAM, which is indexed by pre-synaptic neuron and therefore is not easily accessible for a fast update.
2. A spike packet is delivered to the post-synaptic core as soon as it is emitted, and biological delays (stored in SDRAM as well) are re-introduced by the core modeling the post-synaptic neuron *after* the relevant information has been retrieved from memory; the delay itself is stored into memory, and can be different for different post-synaptic neurons on the same core (Jin et al., 2010a). The weight value is stored in a circular buffer which rotates with the timer event interval, and lumps all the synaptic contributions for one millisecond in a way similar to that described in Morrison et al. (2005). The consequence of delaying the input into the future is that when a synapse is processed, the state of the post-synaptic neuron (e.g., its membrane potential or the presence of a post-synaptic spike) is not available.

The *Deferred Event Driven Model* (DED) for computing plasticity was introduced in Jin et al. (2009) to circumvent these problems. DED enables computation of STDP at the time when a pre spike is received by deferring the weight update process into the future, until enough information is gathered. Post spikes are collected in a spike window, stored in the local core memory, while pre spikes are stored in SDRAM, along with the rest of the synaptic information. Upon the retrieval of the weights related to a pre spike, these two time windows are compared and weight update is performed. Plasticity is therefore always computed on the *next* pre spike arrival, and only if enough time has passed, to guarantee that all the necessary information is available. This poses restrictions on the pre-to-post firing rates: if a pre-synaptic neuron fires with a low rate, the spike information of the post-synaptic neuron might have already expired. Thus, the algorithm loses a pre-post spike pair, even if they were close in time, if the next pre spike arrives after the expiration of the post-spike window. Furthermore, because the algorithm needs to check every spike pair, its efficiency depends on the length of the history and on the number of the pre-post pairs. Such limitations are discussed in Jin et al. (2010b), where the trade-offs between spike-history, efficiency and correctness are analyzed.

Davies et al. (2012) try to address the problem from a different angle, using the *Time To Spike* (TTS) strategy: STDP is computed only upon the reception of a pre spike, using the current membrane potential as a predictor of future spiking activity. By doing so, weight updates can be performed while synaptic information is in local memory, addressing the first of the two problems mentioned above. However, as mentioned earlier, spikes are delivered to the post-synaptic neurons as soon as they are emitted, and the biological delays are reintroduced at the post-synaptic end. This creates errors when using delays, as reported in the original work presenting the TTS approach: the membrane potential used as a predictor of the post neuron firing is the one corresponding to the time of spike *emission* by the pre neuron, rather than that of spike *reception* by the post neuron (after the propagation delay). Such problem makes the TTS algorithm usable and efficient when delays are constant and short, but cannot deal correctly with longer delays. This also creates problems for detecting temporal patterns where delays play an important role (Izhikevich, 2006), such as in the experiments in Section 6.2.

### 3.2. The dedicated plasticity core approach

The previous implementations of plasticity are not limited by the SpiNNaker hardware, but rather by their software implementation. Therefore, we present an alternative approach: instead of having a single core evaluating neural dynamics and plasticity, we divide the job into two parallel processes. One core performs the neural updates and spike integration, while the second core deals with plasticity (see Figures 2C,D). Plasticity operates as a slower process in the background. It processes the whole synaptic block in SDRAM and the information about spike timing, and modifies the weights according to the chosen plasticity mechanism. The proposed approach takes inspiration from previous work where plasticity effects are accumulated and evaluated periodically (Izhikevich, 2006; Nageswaran et al., 2007; Fidjeland and Shanahan, 2010; Pfeil et al., 2012). Plasticity is thus updated less frequently than neural dynamics, which is radically different from the previously described DED model on SpiNNaker.

In our novel approach, the PACMAN mapping tool automatically instantiates a *twin* plasticity core alongside each neural core whenever it detects a neural population with incoming plastic connections. Neural and plasticity cores have access to the same portion of SDRAM through replication, in their local memories, of the look-up tables used to index it. The neural core performs the usual operation that a non-plastic core would perform, thus eliminating all the overheads required by the DED model. The neural core is also in charge of trivially updating a bitmap pre-spike window whenever a pre spike is received, as shown by the dashed arrow in Figure 2C. The plasticity core is concerned solely with the weight update process, which can be performed by walking the local SDRAM weight matrix and computing plasticity at a slower pace. When a neuron in the neural core emits a spike, the corresponding packet is delivered to the plasticity core, and to the post-synaptic neurons as under normal conditions. Because the plasticity and neural core always reside on the same chip, this process does not add overhead to the routing process. This allows to keep track of the post-synaptic spiking history. Here we decided to update the weights every 128 ms and store the spike times with a resolution of 2 ms, as a compromise between performance, platform-specific limitations and precision. Pre-synaptic spikes are stored at the beginning of each synaptic row as spike-history bitmaps. The plasticity process needs to know all the spikes which happened in its considered 128 ms window. This data has been stored by the neural core in one of the spike windows (0 or 1 in the Figure) during the previous 128 ms before the update. For the plasticity core to be able to read this buffer while the neural core is storing the next 128 ms of spikes, we use a double buffer technique: when the plasticity core is reading spike window 0, the neural core is storing the spikes in spike window 1 and viceversa. This has been emphasized in the Figure 2C by using different color codes for the two different processes. The double buffers contain data for different time slots and therefore do not need to be accessed concurrently by the neural and plasticity core, so there is no need for mutual exclusion or locks. Memory contention is eliminated by the fact that the neural core operates in the *current* 128 ms window, while the plasticity core works in the *previous* 128 ms time window. The same technique used for the spike windows could be used on the whole synaptic matrix to ensure coherency of the whole matrix during the entire simulation. Because this method only switches the pointer used to lookup the data between consecutive plasticity periods, this would not change the approach or performances. Whenever a portion of memory is ready for computation, the request for the next row of the synaptic matrix is issued and weight updates of the current synaptic row are performed, thus masking memory access costs through parallelization. This separation of neural and plasticity operations gives rise to an environment where weight update rules can be easily programmed separately. This leverages the reprogrammability of the general processors used by SpiNNaker and the generality of the event-driven API presented in Sharp et al. (2011). The general infrastructure for the framework is presented in Appendix A. While it is worth noting that the difference between neural and the plasticity processes is only in the software running on the ARM cores, they can be thought of as hardware threads. The SpiNNaker software infrastructure does not support threads. If software threads were available, besides the costs related to thread switching, the neural and synaptic update threads would need to split between them the limited local memory (DTCM) and the processor cycles. In SpiNNaker, clock cycles are also limited in order to meet real-time targets. The proposed solution, on the other hand, uses hardware threads (cores), one for neural update and one for synaptic update, with each thread owning all of its local resources. This results in a more efficient use of the available resources. In fact, depending on the relative complexity of the neural and synaptic update processes, the ratio of hardware threads can be adjusted, using *N* neural update for every *M* synaptic update threads (cores). The plasticity core has access to the pre- and post-synaptic spike activity history of the previous 128 ms time window; the first is stored in SDRAM and the second one in DTCM. Such information can be used to compute rates, traces, timing differences or other required variables for different learning rules, as shown by the three rules implemented in this paper.

## 4. STDP

**Figure 3:**
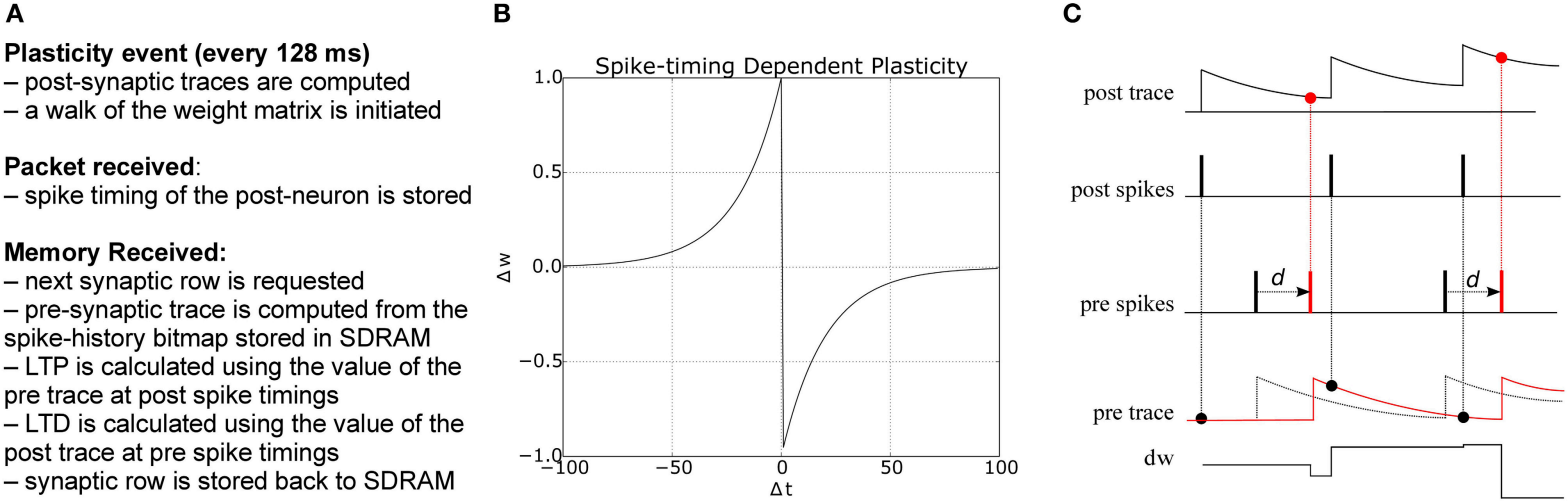
**(A)** Algorithm for STDP learning implementation on the plastic core **(B)** STDP function **(C)** Implementation of pair-based STDP with local traces and delays, as suggested by Morrison et al. (2008): potentiation occurs at post-synaptic spike times and corresponds to the value of the pre-synaptic trace; conversely, depression happens at pre-synaptic spike times and corresponds to the value of the post-synaptic trace. *d* represents the delay, reintroduced at the post-synaptic end; black and red lines represent the traces and spike timings when the delay is reintroduced (red) as opposed to using the presynaptic spike time as reference (black).

Derived from biological observations that synaptic plasticity depends on the relative timing of pre- and post-synaptic spikes (Markram et al., 1997; Bi and Poo, 1998), Spike-Timing Dependent Plasticity (STDP) (Gerstner et al., 1996; Song et al., 2000) has become a popular model for learning in spiking neural networks. In its standard form, STDP weight-updates are expressed by the double-exponential form

(1) $$F(\Delta t)=A_{+}e^{\frac{\Delta t}{\tau_{+}}}\;\Delta t<0$$

(2) $$F(\Delta t)=-A_{-}e^{\frac{-\Delta t}{\tau_{-}}}\;\Delta t\geq 0$$

where Δ*t* = *t*_pre_ − *t*_post_ is the time difference between a pair of pre- and post-synaptic spikes, *A*_+_ and *A*_−_ are scaling factors for potentiation and depression, and τ_+_ and τ_−_ are the time constants of the plasticity curves. The weight update rule is illustrated in Figure 3. There are different strategies for computing the total amount of weight change after seeing multiple pre- and post-synaptic spikes (Morrison et al., 2008), e.g., by considering only nearest neighbor spike pairs, or summing the weight changes *F*(Δ*t*) for all pairs. Here we adopt a form of STDP proposed by Morrison et al. (2008) to compute the weight change using local variables in the form of pre- and post-synaptic traces. Each trace *x_i_* has the form

(3) $$\frac{dx_{i}}{t}=-\frac{x_{i}}{\tau }+A\sum_{t_{i}^{f}}\delta (t-t_{i}^{f}),$$

where *x_i_* is the value of the trace for neuron *i*, *A* is the amplitude by which the trace increases with each new spike at time *t^f^_i_*, and τ is the exponential decay time constant. The concept is illustrated in Figure 3: potentiation occurs at post-synaptic spikes, using the value of the pre-synaptic trace as the weight increase; conversely, depression happens at pre-synaptic spike times, and reduces the weight by the value of the post-synaptic trace.

### 4.1. Methods: implementation of STDP on the plasticity core

The plasticity core is in charge of computing all traces, using the spike timing information collected during the simulation. Weight changes are then computed by walking through all the synaptic block. The pre-trace is computed every time a portion of memory is received through a DMA process using the information in the spike window, while the post trace is computed at the beginning of each plastic phase starting from the spike history bitmap collected during the packet received callback. Traces can have longer time scale than the plasticity window, as the exponential filtering is updated at the beginning of each phase, and the previous value of the exponential filter carries over from one plasticity window to the next. Delay needs to be reintroduced at the post-synaptic end, and can be used to compute the amount of shift required to correctly compute weight de/potentiation, as shown in Figure 3, where the black part shows the spike timing and traces using the presynaptic spike time as the reference, while the red part shows how this reference is shifted once delay has been reintroduced. Not considering the delay generates substantial errors in the weight update. A pseudocode version of the algorithm is presented in Appendix B.

**Figure 4:**
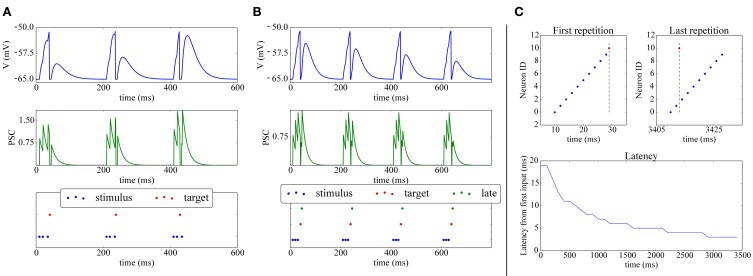
**Shift of post-synaptic firing onset via STDP. (A)** Potentiation: The spike raster plot (bottom) shows that at the beginning of the stimulation 3 input spikes (blue) are needed to make the target neuron (red) fire; after 400ms, potentiation has made the synapse strong enough so that the post-synaptic neuron fires after only 2 spikes. This is also visible in the membrane potential (top) and post-synaptic currents (PSC; middle). **(B)** Depression: (bottom) The green neuron is made to spike consistently after the target (red) neuron, hence its weight gets depressed, as can be observed by its decreasing contribution in the membrane potential (top) and PSC (middle). **(C)** Reduced spike latency: at the beginning of the simulation (upper-left panel) 10 spikes from 10 different input neurons (blue) are needed to make the post-synaptic (red) neuron fire; after repeated stimulation (upper-right-panel), potentiation via STDP makes the red neuron fire already after 2 spikes, hence firing closer to the pattern's start, which is also shown by the latency plot (bottom panel).

A simple experiment in which STDP, implemented with the above scheme achieves synaptic potentiation and depression is shown in Figures 4A,B. The final part of the Figure presents a classical experiment where a plastic neuron can reduce the latency of its firing to a repeatedly presented pattern (Mehta et al., 2000; Song et al., 2000): a (red) neuron receives connections from 10 inputs neurons (blue) which fire at 2 ms from each other; during the first repetition all the 10 input neurons are required to make the target neuron fire. After repeated presentations, due to potentiation, only two input neuron spikes are needed to elicit activity in the post neuron, which responds with a lower latency to the onset of the pattern.

### 4.2. Results: pre-post pairing using a teacher signal

**Figure 5:**
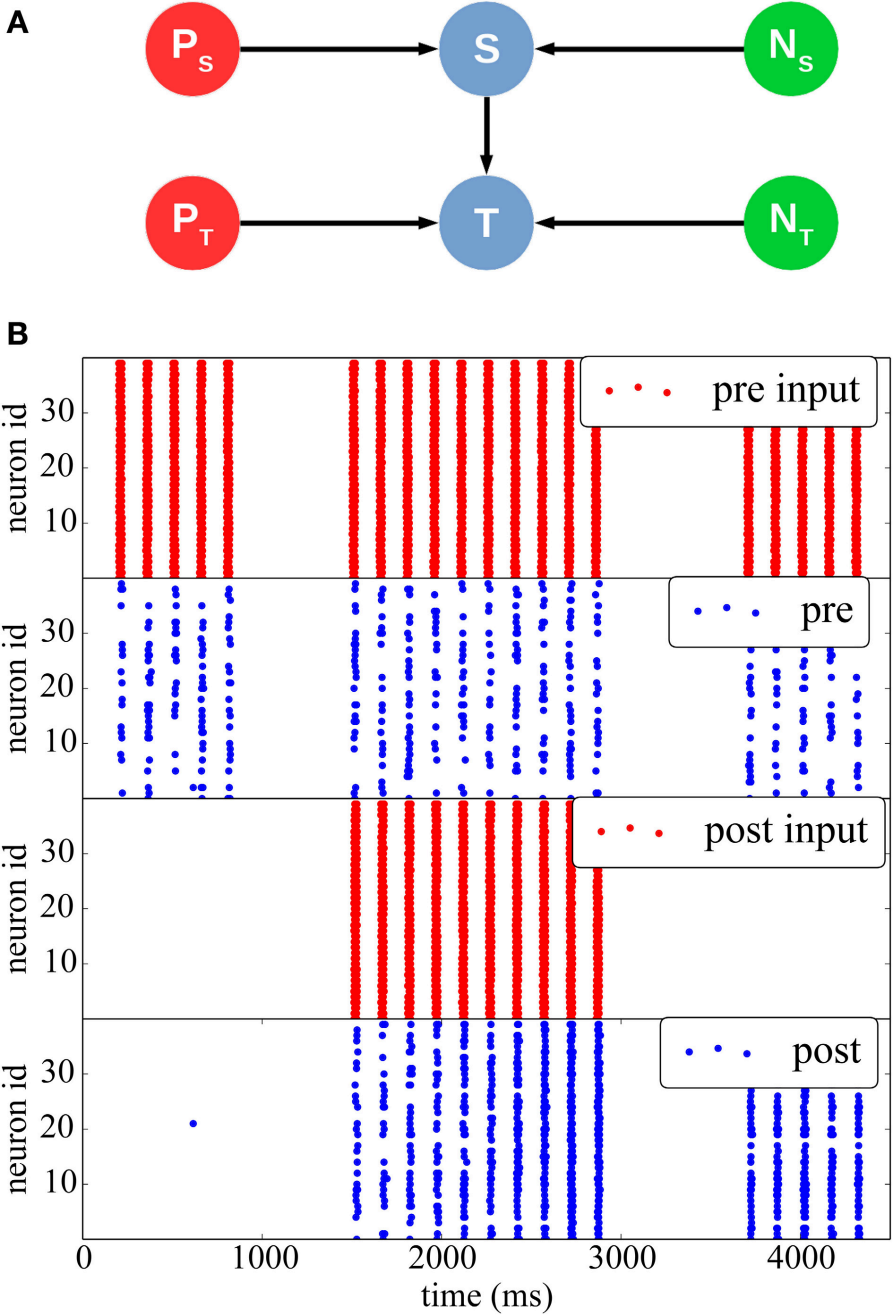
**STDP with a teacher signal. (A)** Network structure: two different Poisson spike sources (red) are used as supervisor signals to individually stimulate the *Stimulus* (top) and *Target* (bottom) populations at different times (blue), which also received noise from two separate sources (green). **(B)** Initially (between 0 and 1000 ms), only the pre-synaptic population is stimulated, but the synaptic weights are weak, thus the resulting spikes (blue) do not elicit post-synaptic spikes. Between 1500 and 3000 ms, both populations are stimulated with a 10 ms time difference, such as to induce synaptic potentiation. The effect can be seen between 3500 and 4500 ms, when the teacher signal for the post-synaptic population is removed: after the potentiation the pre-synaptic spikes are able to drive the post-synaptic neurons by themselves.

In Figure 5 we reproduce results of a classical stimulation protocol for potentiation induced by pre-post synaptic pairing. The network comprises a *stimulus* population and a *target* population, each separately driven by two different Poisson sources emitting spike bursts at high frequency (350 Hz) for short periods of time (20 ms). Both populations also receive independent background noise. The Poisson and noise source populations are interconnected with a one-to-one connectivity pattern to their respective inputs and outputs. The stimulus and the target populations are interconnected with a 50% probability.

At the beginning of the simulation, external stimulation coming from the stimulus population is not strong enough to trigger activity in the target post-synaptic population (0 ≤ *t* ≤ 1500 ms). Afterward (1500 ≤ *t* ≤ 3000 ms) the stimulus and target populations are stimulated together by their respective Poisson inputs, so that the target population spikes 10 ms after the stimulus population, hence inducing potentiation. Finally, for 3500 ≤ *t* ≤ 4000 ms, the Poisson process feeding the post-synaptic population is removed, and the post-synaptic population is only stimulated by inputs from the pre-synaptic population. It can be seen that because of the induced potentiation, the pre-synaptic input is now strong enough to make the target population fire without any supervisor input.

### 4.3. Results: balanced excitation

**Figure 6:**
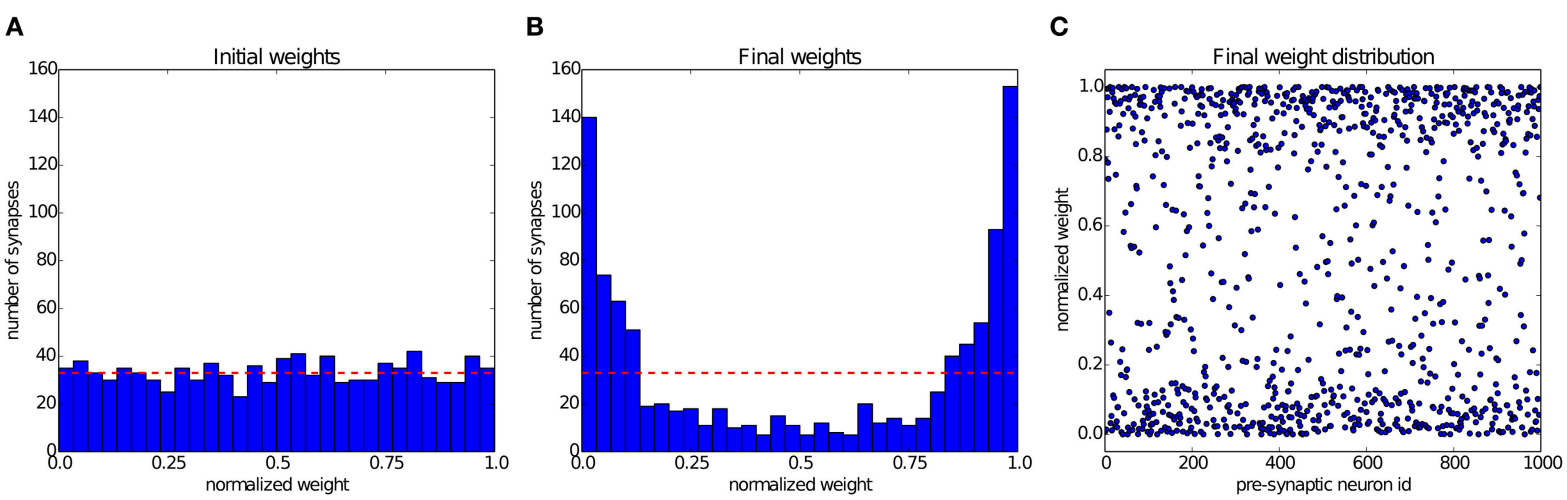
**Competition between synapses undergoing STDP: In the experiment introduced by Song et al. (2000), 1000 uncorrelated pre-synaptic neurons, firing at a Poisson-rate of 20 Hz, project onto a single post-synaptic neuron. (A)** Initial uniform weight distribution before plasticity. **(B)** After 300 seconds of stimulation STDP has divided the synaptic weights into *weak* and *strong* ones, thereby regulating the activity of the post-synaptic neuron. The red line shows the mean of the initial weights. **(C)** Scatter plot of the final weight distribution.

Song et al. (2000) have shown that STDP can establish a state of balanced excitation in the post-synaptic neuron, which makes it more likely to fire with a controled output rate in response to fluctuations in its input. This is achieved by competition between the synapses that project onto the post-synaptic neuron, induced by STDP. The characteristic effect described by Song et al. (2000) is that STDP creates a bimodal distribution of input weights, pushing them either toward the minimum or maximum values, and creating groups of *strong* and *weak* synapses. In Figure 6 we simulate a group of 1000 input neurons, firing independently according to a Poisson process at 20 Hz, and projecting onto a single output neuron. The weights are initialized uniformly, and then undergo STDP. After 300 s of simulation, the distribution of synaptic weights in Figure 6 shows clearly the characteristic separation into two groups of very different strengths. The experiment can be observed in Movie 1 (Supplementary Material), which shows the weight distribution as the simulation is running.

## 5. BCM

**Figure 7:**
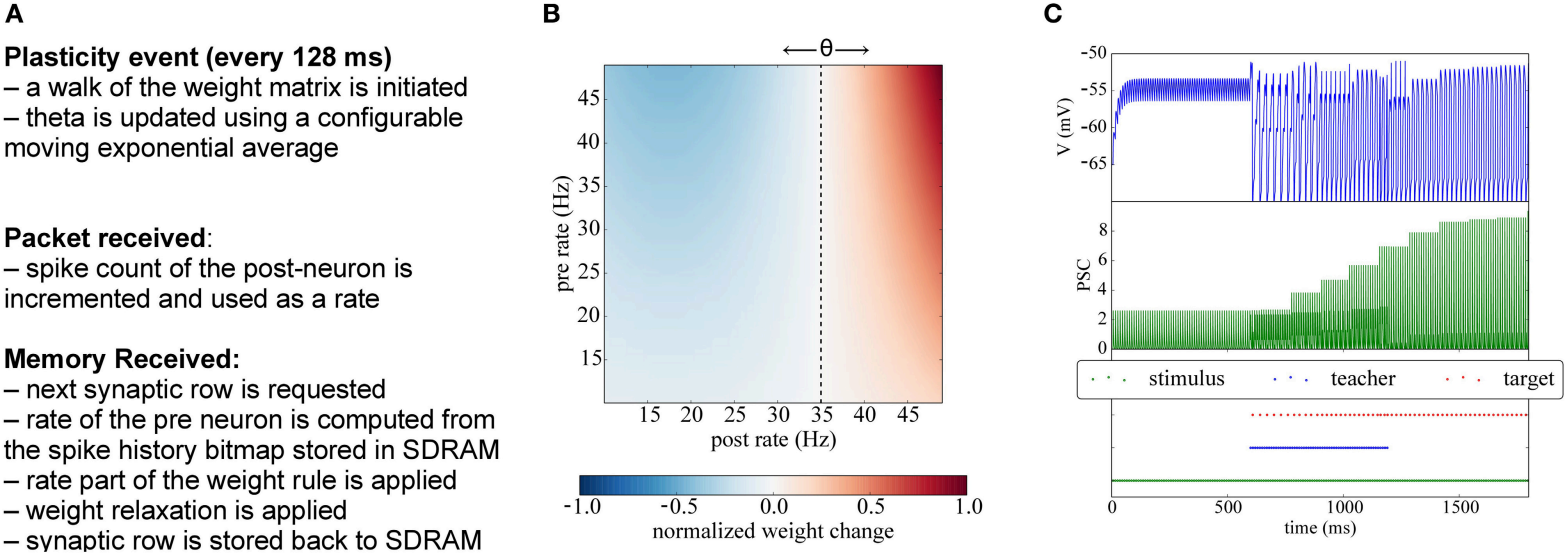
**Implementation of BCM plasticity. (A)** Algorithm for BCM learning on the plasticity core. **(B)** Illustration of the BCM rule: normalized weight change as a function of the pre- and post-rates, with θ = 35 Hz. **(C)** Potentiation experiment using a teaching signal: when stimulation is paired with a teacher signal that forces the post-synaptic neurons in the target population to fire, the weights get potentiated and become strong enough to drive the post-synaptic neuron.

The *BCM rule*, named after their inventors Bienenstock, Cooper, and Munro (Bienenstock et al., 1982), is a rate-based synaptic plasticity rule, introduced to model binocular interactions and the development of orientation selectivity in neurons of the primary visual cortex. The BCM rule is based on Hebbian principles, but introduces synaptic competition by correlating the pre-synaptic rate with a non-linear function of the post-synaptic rate. In its simplest form the BCM rule computes this non-linearity as the product of the post-rate with its deviation from the mean post-synaptic activity (see Figure 7B):

(4) $$\frac{dw}{dt}=[r_{post}(r_{post}-\theta )r_{pre}]\delta -\epsilon \cdot w\;\;.$$

Here *w* denotes the synaptic weight and *dw*/*dt* its change, *r_post_* and *r_pre_* are the firing rates of the pre- and post-synaptic neurons, θ is the modification threshold, which is computed here as the mean firing rate of the post-synaptic neuron, δ is a learning rate, and ϵ a weight-decay parameter. If *r_post_* exceeds the mean firing rate θ, the weight is potentiated; conversely, for lower activity (*r_post_* < θ) the weight is depressed. The learning rate parameter δ can be used to normalize the magnitude of the synaptic weight change according to the neural model used. Many variations of the BCM rule have been studied since its introduction, using different kinds of non-linearities, but here we study only the basic version from Bienenstock et al. (1982).

### 5.1. Methods: implementation of BCM on the plasticity core

Since the BCM rule only requires firing rates, the plasticity core just has to increment a counter whenever a post-spike is received, and to use a low-pass filtered version of the rate. Analogously, when processing a row relative to an afferent neuron, the number of spikes received during the previous phase is used to update the pre-synaptic rate information. At the end of each plasticity phase θ (the threshold parameter representing the mean rate) is updated using a configurable exponential moving average, and the pre spike windows are reinitialized. A pseudocode version of the algorithm is presented in Appendix C and is outlined in Figure 7A.

In Figure 7C we show a classical potentiation protocol using the BCM rule. For the first 600 ms the target population is only receiving spikes from the stimulus population, but the weights are too weak to cause firing in the target population. Between 600 and 1200 ms, a teacher population is activated which is strong enough to drive the target population, thereby potentiating also the simultaneously active stimulus-target connections. Afterwards, when the teacher population is switched off, the stimulus population alone is able to drive the target population without teacher input.

### 5.2. Results: emergence of orientation selectivity with BCM

The BCM rule has been originally proposed in Bienenstock et al. (1982) to explain how neurons in the primary visual cortex can acquire their feature selectivity from sensory stimulation. As a test of our implementation of BCM on SpiNNaker we replicate a simple neural network with lateral inhibition which undergoes plasticity while receiving monocular visual input in the form of oriented bars.

The network consists of 2 layers, an input layer which comprises 16 × 16 neurons and an output layer with 4 neurons. Each neuron in the input layer projects, in an all-to-all fashion, to the output neurons. All synapses are initialized with random weights and delays. Each neuron in the output layer has an inhibitory projection to every other neuron, forming a network of lateral antagonism (Shouval et al., 1997). The aforementioned connectivity pattern matches anatomical data, for example the *lateral plexus* of the Limulus's eye, as originally found by Hartline et al. (1956). Bienenstock et al. (1982) themselves point out that no selectivity is achieved without lateral inhibition.

For this experiment four images of oriented bars are used as input stimuli, each rotated by 45°C. Bars are 3 pixels thick and 12 pixels in length, and the intensity of each pixel is a random value between 0.8 and 1.0. Each pixel is converted to Poisson spike trains, in order to simulate spikes coming from the retina or LGN. The firing rates are proportional to the value of the pixels, while all firing rates are scaled such that the input layer generates approximately 1000 spikes per second. During the simulation each orientated bar is presented to the network in a random order for 1 s and for 80 repetitions. Learning takes place in the synapses between the input and output layer, while the inhibitory synapses in the output layer are static and set to a weight of −9 nA.

**Figure 8:**
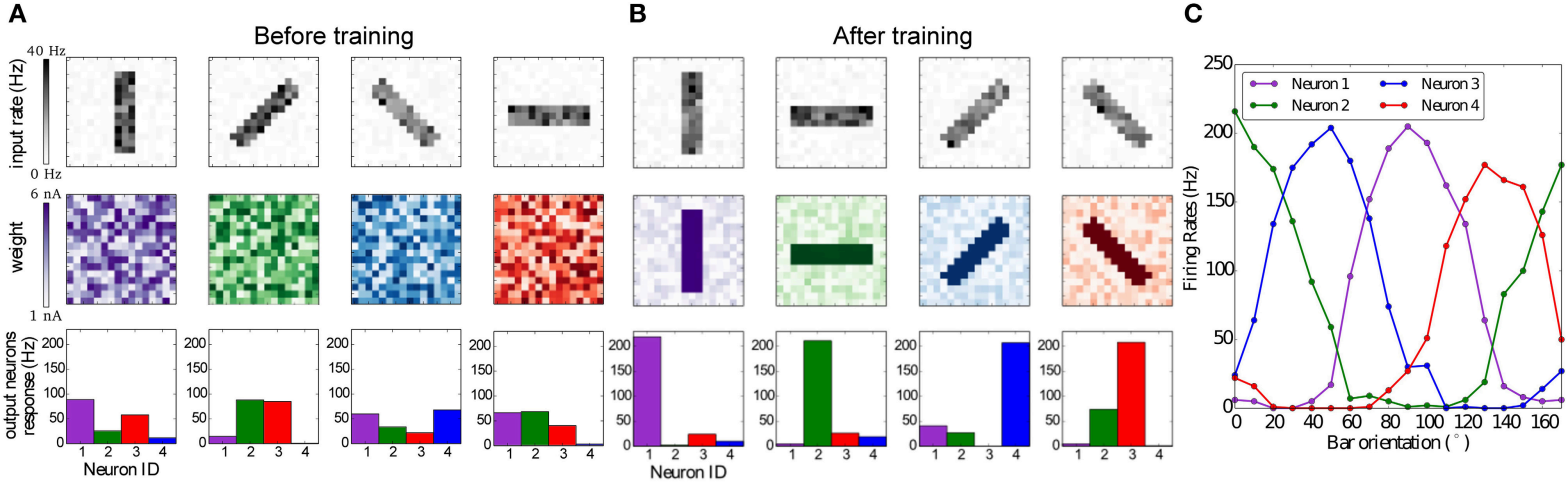
**Emergence of orientation selectivity with the BCM learning rule. (A,B):** The top row represents the input stimuli bars presented in different orientations, the total firing rate for each stimulus is 1,000 Hz; the middle row shows the weight matrix for the output neurons, with **(A)** random initial weights and **(B)** results after the training, where it can be observed that neurons have developed their receptive fields according to the input stimulation; the bottom row shows the firing rates of the output neurons, where each color codes for a different neuron which has learned a preferred orientation. **(C)** Orientation tuning curves obtained by rotating a horizontal bar counter-clockwise with a step size of 10°C.

The results are summarized in Figure 8. Figure 8A shows that the weights and neuronal responses to input stimuli are initially random. At the end of the simulation, Figure 8B shows that each output neuron has developed via BCM plasticity a receptive field that corresponds to one particular orientation. In Figure 8C we show the orientation tuning curves of each neuron, measured by rotating the stimulus bar counter-clockwise in 10°C steps. The results show that each neuron has successfully learned to respond best to one preferred orientation, which is in line with previous modeling studies and experimental and anatomical data (Moore and Freeman, 2012; Jeyabalaratnam et al., 2013). The learning can be observed in Movie 2 (Supplementary Material), where the four receptive fields are emerge from the repeated presentation of the input stimulation.

## 6. Voltage-gated STDP

Brader et al. (2007) have presented an STDP rule that is triggered by the arrival of pre-synaptic spikes, and in which the change in synaptic efficacy is a function of post-synaptic depolarization and of an internal variable at the spike arrival time. The rule is motivated by the necessity to design learning rules which are at the same time biologically plausible, but also compatible with implementation constraints on neuromorphic devices. Several studies have demonstrated the ability of the learning rule to discriminate complex spatio-temporal patterns (Indiveri and Fusi, 2007; Giulioni et al., 2009; Mitra et al., 2009), even if the synapses are allowed to take on only one of two stable states. Every time the post-synaptic neuron emits a spike an internal variable *C*(*t*), representing calcium concentration due to back-propagating action potentials, is incremented by a value *J_C_* and then decays with a time constant τ_*C*_ according to the dynamics described by

(5) $$\frac{dC(t)}{dt}=-\frac{C(t)}{\tau_{C}}+J_{C}\sum_{i}\delta (t-t_{i}),$$

where *t_i_* are the post-synaptic spike times. Potentiation and depression happen only if *C*(*t*) is in an appropriate interval [θ*^h^_down_*, θ*^h^_up_*] for potentiation and [θ*^l^_down_*, θ*^l^_up_*] for depression. Post-synaptic membrane depolarization *V*(*t*) influences this plasticity rule, triggering potentiation (or depression) only if the membrane potential of the post-synaptic neurons is higher (lower) than a threshold value θ_*V*_ at the time of arrival of a pre-synaptic spike (*t_pre_*). Modification of the synaptic efficacy *w* can then be summarized by the following equations:

(6) $$w=w+a\text{ if }V(t_{pre})>\theta_{V}\text{ and }\theta_{down}^{h}\leq C(t_{pre})<\theta_{up}^{h}$$

(7) $$w=w-b\text{ if }V(t_{pre})\leq \theta_{V}\text{ and }\theta_{down}^{l}\leq C(t_{pre})<\theta_{up}^{l}$$

where *a* and *b* represent the constant weight increase and decrease values respectively.

If none of the conditions in (6) and (7) are met, or if no spike is received in the period of time considered, then the weight drifts toward one of two stable values (*w_min_* and *w_max_*). The direction of the drift is determined by comparing the current weight *w* to a threshold θ_*W*_, and speed of the drift toward the minimum and maximum stable states is determined by the constants α and β respectively. This leads to the following dynamics:

(8) $$\frac{dw(t)}{dt}\;=\alpha \text{ if }w(t)>\theta_{W}$$

(9) $$\frac{dw(t)}{dt}\;=-\beta \text{ if }w(t)\leq \theta_{W}$$

### 6.1. Methods: implementation of voltage-gated STDP on the plasticity core

The voltage-gated STDP rule needs further information from the post-synaptic neuron, as the membrane potential gates potentiation or depression. The cores communicate this information as means of shared memory in SDRAM, using a double buffer technique so that they always work on different phases. This induces a slight overhead in the neural core, which has to perform the check against θ_*V*_ and saves the result for each millisecond in a bitmap stored in memory. The plasticity core retrieves the results of the comparison at the beginning of each plasticity phase, and uses them in the weight update process. At the same time the function *C*(*t*) is computed starting from the post neuron spike timings, similarly to computing the STDP traces. A pseudocode version of the algorithm is presented in Appendix D.

**Figure 9:**
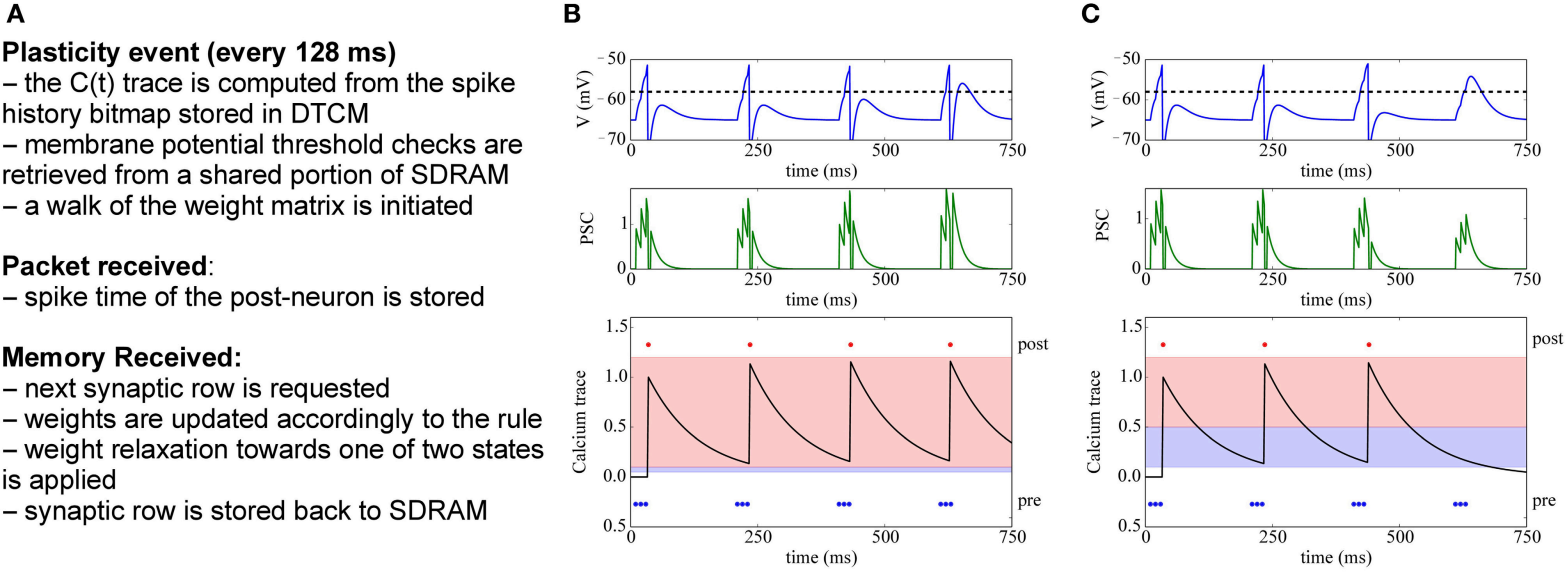
**Implementation of voltage-gated STDP. (A)** Concept for implementing voltage-gated STDP on the plasticity core. **(B)** Example of synaptic potentiation: three pre-synaptic spikes (blue) arrive while the membrane potential is greater than θ_*V*_ and θ*^h^_down_* ≤ *C*(*t_pre_*) < θ*^h^_up_* (red-shaded area in bottom row). Initially, the post-synaptic neuron (red) fires after the third spike, after potentiation only two spikes are needed to make the target neuron fire. **(C)** Depression example: three pre-synaptic spikes (blue) arrive while the membrane potential is less than θ_*V*_ and θ*^l^_down_* ≤ *C*(*t_pre_*) < θ*^l^_up_* (blue-shaded area in bottom row). After depression takes place, the post-synaptic neuron (red) no longer fires after receiving the 3 input spikes.

The basic dynamics of this voltage-gated STDP rule are shown in Figure 9: The bottom row shows the trace of the calcium variable *V*(*t*), which is increased by *J_C_* whenever the post-synaptic neuron fires, and then exponentially decays with time constant τ_*m*_. The central part shows the potentiation of a synapse, because here the pre-spikes arrive when *V*(*t_pre_*) > θ_*V*_ and θ*^h^_down_* ≤ *C*(*t_pre_*) < θ*^h^_up_*, and thus fewer spikes are needed to drive the target neuron. Conversely, on the right we observe the depression of a synapse, because pre-spikes arrive when *V*(*t_pre_*) ≤ θ_*V*_ and θ*^l^_down_* ≤ *C*(*t_pre_*) < θ*^l^_up_*. After depression, the synaptic input is too weak to make the target neuron fire.

### 6.2. Results: learning temporal patterns

To verify our implementation of the voltage-gated STDP rule by Brader et al. (2007), we implemented the model by Coath et al. (2013) for learning temporal structures in auditory data, which has originally been implemented on a neuromorphic chip in Sheik et al. (2012). The study focused on learning dynamical patterns in the context of a sound perception model by tuning auditory features through presentation of stimuli and learning using the STDP rule implemented in VLSI.

**Figure 10:**
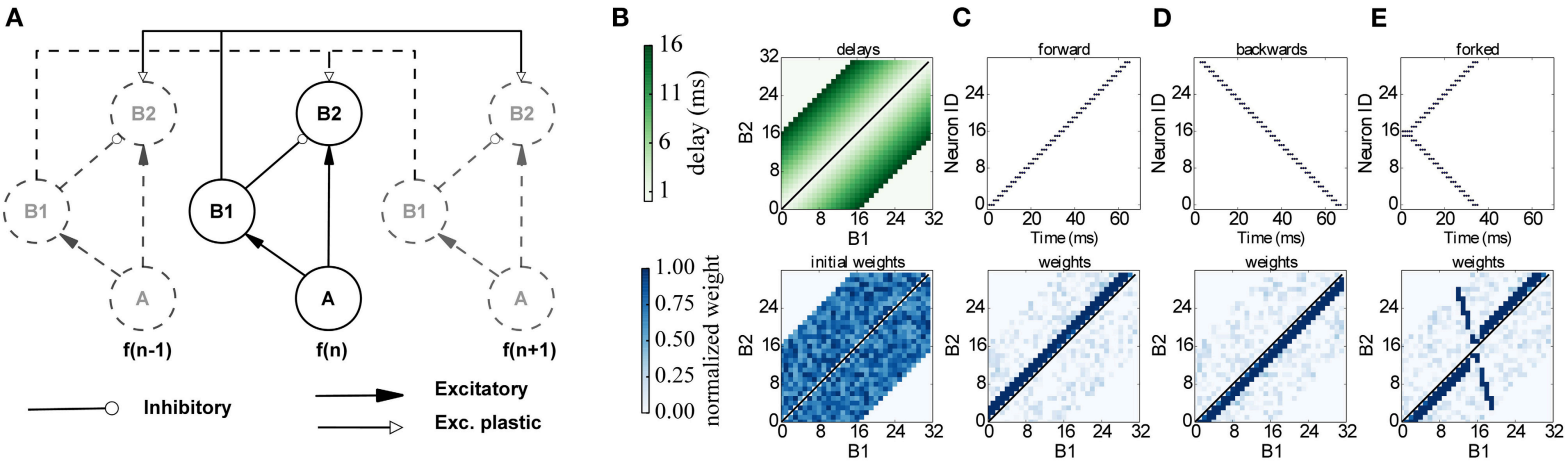
**Learning temporal patterns with the model by Coath et al. (2013) and Sheik et al. (2012). (A)** Network structure: each frequency channel *f* comprises three neurons *A*, *B*_1_ and *B*_2_, and is connected to other frequency channels (dashed in figure), arranged in a tonotopical way, with distance-based delays and plastic connections. **(B)** Delay matrix (top) and example of initial random weight matrix (bottom). **(C–E)** Input spikes (top) and resulting weight matrix (bottom) after learning for a forward, backwards and forked frequency swipe respectively.

The proposed network learns to respond to particular input timing patterns. The network comprises 3 layers of tonotopically organized frequency channels, representing different positions on the basilar membrane. The first layer *A* represents a spiking signal produced by an artificial cochlea (such as the one in van Schaik and Liu, 2005); each neuron in the *A* layer projects to a neuron in 2 layers, *B*_1_ and *B*_2_ through excitatory synapses, while *B*_1_ projects to *B*_2_ through inhibitory synapses. Each neuron in *B*_2_ also receives plastic connections from all the neighboring *B*_1_ neurons, with delays proportional to the distance, as shown in Figure 10. Since delays are programmable in SpiNNaker we incorporated them directly in the *B*_1_ to *B*_2_ connection, and not through a separate neural population as in the original model. This delay property is essential for learning: correlation between the delayed feedback arriving from other *B*_1_ neurons to the *B*_2_ neurons is detected by the plasticity rules, and it controls synaptic potentiation and depression by coincidence detection. To implement the model on SpiNNaker while coping with the 1 ms time resolution used in the current neural kernels we multiplied all the time quantities by 10. For learning we use the same three input patterns that were used in the original model (see Figure 10): Pattern (C) is a forward frequency sweep, where every frequency (and therefore every *A* neuron) is activated in order, with a short delay between one presentation and the next. For pattern (D) we perform the same frequency sweep, but we move backwards through the frequency space. Finally for pattern (E) we perform a forked frequency sweep, starting from the middle frequency. We present the stimulus multiple times to the network and analyze what it has learned by examining the *B*_1_/*B*_2_ weight matrix. The results are presented in Figure 10, and can be compared with the results in Figures 7, 8 in Sheik et al. (2012). After repeated presentations of the target patterns, the weight matrix, initialized randomly, converges to a state where it is only sensitive to the spike-timing pattern presented, by coincidence detection through delay lines. The emergence of the connectivity matrix for the forked frequency sweep can be observed in Movie 3 (Supplementary Material).

## 7. Performance analysis and discussion

In Diehl and Cook (2014), the authors describe an STDP variation of the DED which follows the strategy proposed in Morrison et al. (2008) by storing traces in SDRAM, rather than performing spike pairing as proposed in Jin et al. (2010b). The authors evaluate the performance of their implementation as well as the one present in the stable release of the SpiNNaker software package^1^ in terms of synaptic events processed per second, as done in Sharp and Furber (2013) and Stromatias et al. (2013). They do so by feeding a leaky integrate-and-fire population of 50 neurons with a neural population of variable size that produces spikes at ≈250 Hz, according to a Poisson process, with a 20% connection probability. They report that their implementation of plasticity is capable of handling around 500K synaptic events per second per core (using 150 input neurons), while the original SpiNNaker implementation is limited to 50K events.

We adopt a similar strategy to evaluate our plasticity algorithms, but in more stringent conditions, and with a larger connectivity range. Rather than testing a single core we test a full chip (16 cores). In this way, we can also evaluate the effects of memory contention between different cores, as memory access can be a bottleneck for simulations on SpiNNaker. We model a population of 800 neurons in a single SpiNNaker chip (8 cores modeling neurons and 8 cores dedicated to plasticity) fed by an input Poisson neural population of 150 neurons with a variable rate, and measure the maximum firing rate at which the simulation can run in real time. We take as a starting point the connectivity levels reported by Diehl and Cook (2014) (20% interconnection probability, 150 × 50 × 0.2 synapses, for a total of 1,500 per core and 24,000 per chip if considering 16 cores) and increase the connectivity level up to 100% (7,500 synapses per core, 120,000 per chip). This results in synaptic rows which are 5 times longer, as every pre-synpatic neuron is connected to every post-synaptic neuron in each core, rather than only 20% as in the original experiment. We then scale the model further up by adding more pre-synaptic neurons so as to reach a total of 156,000 synapses. The performance analysis of the algorithms proposed in this work uses the same leaky integrate-and-fire current based neuron. To be able to scale the rate while maintaining the post-synaptic activity constant, we set all the weights and all the weight increments in the plasticity rules to 0, similarly to the approach in Stromatias et al. (2013). This means that plasticity is normally computed, but the weight is clipped to 0 and stored back in SDRAM. Such values are set at runtime and cannot therefore by optimized by the compiler; we have also ensured that setting these values to 0 would not bypass part of the code by removing some optimization tests (like not updating weights which do not change), thus ensuring that the code behaves in our test case as the worst possible real case. Post-synaptic activity is induced by feeding the leaky integrate-and-fire neurons with a current inducing an activity of ≈ 22 Hz.

We check if at any moment any core is lagging behind real time as this would make the simulation incorrect and unrepeatable. We also check if a walk through of the weight matrix is completed before the end of the plasticity period or, in other words, if the plasticity process is finished before the next one starts, as overlapping in this sense is not possible when operating in real time. This allows us to measure the maximum number of synaptic events that can be handled in real time by a single SpiNNaker chip, using the three learning rules proposed in this paper (STDP, BCM and voltage-gated STDP), and to understand if the performance is limited by the neural or the plasticity core.

**Figure 11:**
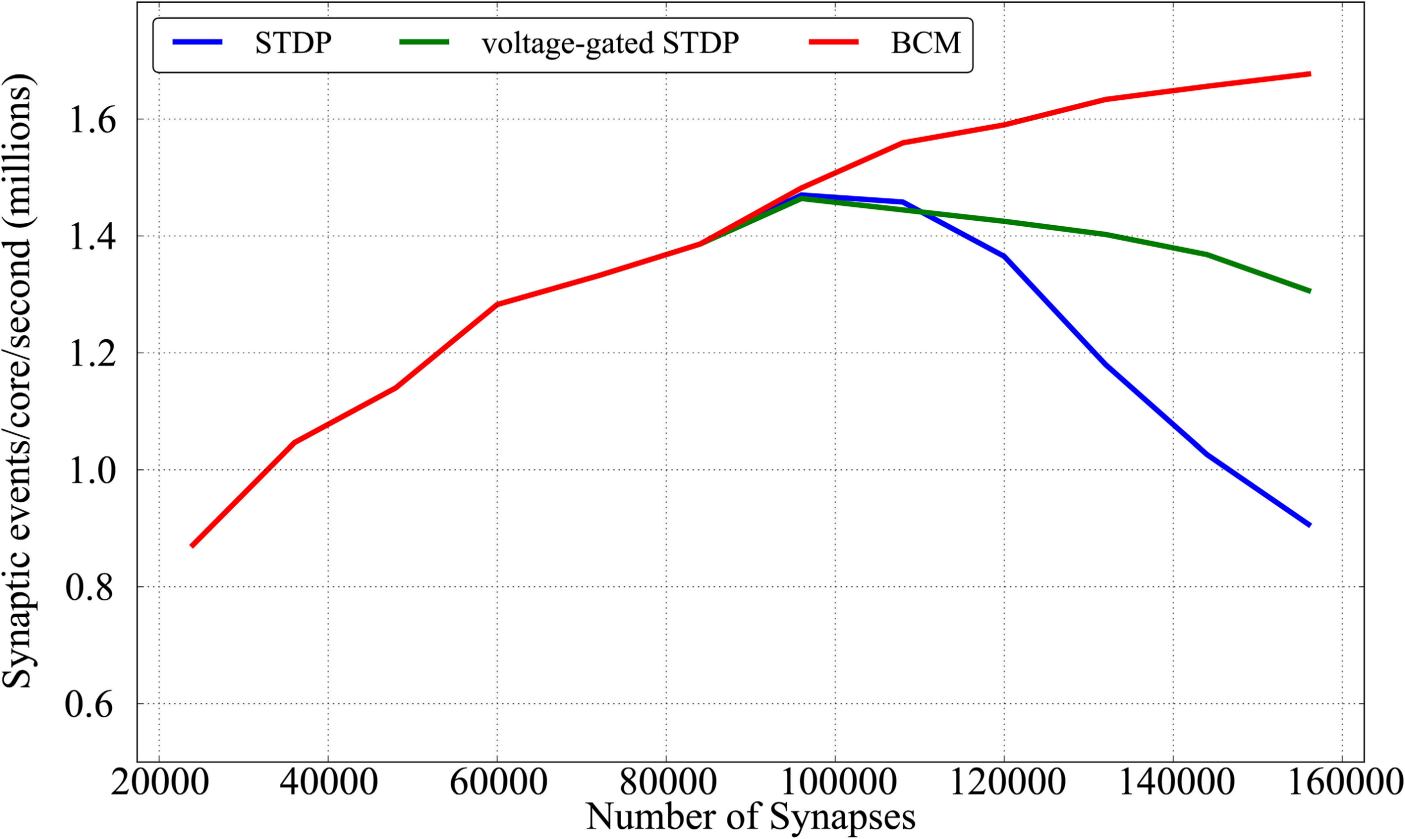
**Performance evaluation of the three learning rules in terms of synaptic event processed per core per second as a function of different number of synapses**.

Results are shown in Figure 11; for each given connectivity level (number of synapses) pre-synaptic firing rates are increased until the limit after which real-time simulation is no more possible. Each point of the plot hence represents the limits of the approach for a given connectivity, for each of the plasticity rules implemented. From the Figure it can be observed that the three learning rules implemented within this framework have similar performances untill the limit of 96,000 synapses (corresponding to scaling up to 80% connectivity the model by Diehl and Cook, 2014). This is due to the fact that, up to that point, all three learning rules are limited by the neural cores lagging behind real time, rather than by the plasticity process taking too long. Such limit peaks just below 1,5 million synaptic events per second per core for all three rules (23 million events for the full chip). In a non-plastic performance analysis, Stromatias et al. (2013) measured a maximum throughput of ≈ 2.38 million synaptic events per second per core. After this connectivity level the complexity of the two STDP models (standard and voltage-gated) becomes the limiting factor, and a complete walk of the synaptic matrix is not possible anymore within the 128 ms period used in this paper. The BCM algorithm is not affected by this, as the algorithm is computationally less intense, and keeps improving above 1,6 M synaptic events per second per core. The decay in performances reflects the complexity of the algorithm considered: standard STDP, being more complex than the voltage-gated version, has a sharper decrease in performances.

When comparing these scenario results with the previous plasticity models based on the DED by Jin et al. (2010b) and Diehl and Cook (2014) (around 50k and 500k synaptic events per second per core respectively in the 20% case - the leftmost part of Figure 11), it must be remembered that these algorithms work with a 1 ms spike-window resolution, while the experiments proposed in this paper have adopted a resolution of 2 ms. Also the former algorithms might lose spikes, while in the approach presented here the contributions from *all* the spikes are accumulated (or, in other words, no spike is lost).

While our approach was designed for maximal flexibility, there might be tradeoffs in terms of efficiency for some scenarios, depending on connectivity and firing rates. One limitation of our approach is, for example, that every plasticity event triggers an update of the complete synaptic matrix. For the rules proposed in this paper is not possible to selectively update only some rows. For pre/post sensitive rules (such as STDP) destinations are encoded in the synaptic row, which is stored in SDRAM, so it is not possible to know if a pre neuron connects to a post neuron which has fired (thus inducing LTP) before retrieving the row itself. In rate based models such as BCM, where the firing rate is considered as a moving average, the absence of spikes is not sufficient to ensure there is no plasticity in act. Finally rules with relaxation toward one (BCM) or multiple (voltage-gated STDP) states require a weight update even in the absence of a spike.

Since in our implementation plasticity updates occur every 128 ms, pre-synaptic firing rates should be at least on the order of ≈ 7 − 8 Hz to avoid having to update silent synapses regularly. In scenarios with lower firing rates, a purely event-driven update would be more efficient. However, a main motivation for our approach is to ensure real-time performance, even in situations with momentarily high load, e.g., if multiple neurons are firing in bursts. Such scenarios are common when using natural inputs with coincident input spikes or models with oscillatory background signals. In such cases the plasticity core approach offers greater flexibility to process plasticity in real time: instead of having to process neural and synaptic updates of all simultaneous spikes within the 1ms time step of the neural core, which might be challenging for complex plasticity rules or for complex neural models, our approach accumulates events over the longer time window of the plasticity core.

This decoupling enables the neural cores to maintain the real-time constraints, and opens up new possibilities for trade-offs to reduce the load on the plasticity cores if necessary. The simplest possibility is, as in the DED model, to lower the number of neurons simulated by each neural core (and therefore also by its associated plasticity core). Other options, although not implemented in the first proof-of-concept presented in this paper, are possible. For the initial results presented in this work we maintain a 1:1 ratio between neural and plasticity cores, but this will likely not be optimal for all scenarios. When looking at Figure 11 it can be see that the two STDP models show a sweet spot for performance at around 80% connectivity. Before such maximum the performance is limited by the neural cores, while after that is the plasticity core which is not able to keep up with the real-time requirements. An interesting alternative would be to allocate more plasticity cores to a single neural core, and adapt the *plasticity:neural* core ratio according to the network characteristics and to the computational complexity of the neural and plasticity algorithms and the associated workload.

A limiting hardware factor for any implementation of plasticity on SpiNNaker is the memory bandwidth, because rows of the synaptic matrix need to be written back to SDRAM. It was shown in Figure 9 of Painkras et al. (2013) that writing is the main bottleneck, since the read bandwidth is twice as high. Our approach reduces the write load, since rows are only written back to SDRAM at most once every plasticity interval, rather than once every pre-synaptic spike as in the DED model. This means that, for example, if pre-synaptic neurons are firing at 24 Hz each synaptic row would be transferred back to SDRAM 24 times per second using the DED model, but only 8 times with our approach.

Finally another possibility is to increase the duration of plasticity intervals, which increases the time available for computing the updates, but comes at the cost of larger memory requirements for storing traces in the core-local DTCM. For long plasticity intervals this might grow beyond what can be stored in DTCM (64 Kbytes for each ARM core, of which some space needs to be reserved for other parameters and buffers). The capacity can be increased by lowering the precision for storing the traces, or using a coarser time resolution. All these possible trade-offs, although not fully explored in this initial work, show the versatility of the approach, which can be adapted to different situations and modeling needs, and constitutes one of its key features, as discussed in the last Section.

## 8. Discussion

Current research on understanding the relationship between the local electrochemical processes of synaptic plasticity and their manifestations as high-level behavioral learning and memory is increasingly relying on theoretical modeling and computer simulations (Gerstner et al., 2012). Because of the great diversity of plasticity phenomena observed in biology and the resulting diversity of proposed mathematical models, as well as the computational complexity of spiking neural network simulations dominated by the costs of synaptic processing, it is necessary to create simulations tools that provide both the flexibility to try out new models easily, and the speedup of specialized hardware. This meets the demand of increasingly large neural network simulations, both for studying brain function, and for applications in artificial intelligence (Le et al., 2012). SpiNNaker has proven to be a well-suited platform for massively parallel large-scale simulations of spiking neural networks, and is flexible enough to let researchers implement and test their own computational models in standard programming languages. The previous Deferred Event Driven Model of handling events in SpiNNaker has made it difficult to implement plasticity rules with arbitrary triggering events (pre-, or post-synaptic, or at regular time intervals), rules which depend on third factors available only at the post-synaptic neuron, or plasticity in networks with variable axonal delays. We have presented here a framework which uses the modular architecture of SpiNNaker and delegates weight updates to dedicated plasticity cores, while the network simulation operates on the remaining neural cores. We have shown that a variety of commonly used plasticity rules can be exactly replicated on this framework, with a greatly increased capacity of processing plasticity events in real-time, by running experiments on a 4-chip SpiNNaker board. The separation of neural from plastic concerns is the feature that enables the great flexibility of the architecture. The two cores work in parallel on different time scales and phases, and the plasticity core has all the information to compute plasticity for the recent past, can access the weight matrix shared with the neural core, and any other information that can be passed through means of shared memory, e.g., membrane potentials and spike timings of the pre- and post-synaptic neurons. All this information can be pre-processed before plasticity is computed, which allows e.g., the computation of rates in an otherwise spike-based simulation. The architecture can be configured easily, using PyNN scripts. This standard, high-level neural language makes it easy to integrate and explore new learning rules into the SpiNNaker architecture.

The approach presented in this paper is tailored to SpiNNaker and to its specific architecture, design and constraints. Nonetheless the same principles could be applied to other digital-analog hybrid architectures, where efficient neural simulation could be realized on one neuromorphic chip, whereas complex plasticity rules could be realized off-chip on computers or FPGAs. Regarding GPUs it appears to be more favorable to let each kernel perform the same operation following the SIMD paradigm. Fidjeland et al. (2009) sequentially use two different kernels, one for neural updates and one for applying plasticity updates. Such kernels do not run in parallel on the same GPU, but serially. This does not constitute a problem when running accelerated simulations, which is the common case for GPUs, but can raise difficulties when running in closed-loop real-time scenarios, as in neurally inspired robotics (Galluppi et al., 2014). In fact concurrent kernel execution is a feature that has only recently been introduced in GPUs, with the NVIDIA Fermi architecture. Using such technique, a plasticity and a neural kernel could be instantiated concurrently on the same GPU, in a similar way to what is done in our approach. Memory access patterns, and the possibility of accessing contiguous portions of memory is a key factor when programming a GPU (Brette and Goodman, 2012). It could be speculated that applying an approach like the one proposed in this paper would have the benefit of guaranteeing memory coalescence, as the synaptic matrix is sequentially accessed when walking through it. Multi-core or cluster architectures could also in theory benefit of separating neural simulation and plasticity, running either on different threads or on different cores, and with different time scales. However, clusters are equipped with more powerful processing units than SpiNNaker, so computing neural and synaptic updates in different cores could introduce unnecessary overheads and synchronization difficulties, particularly regarding memory bandwidth and access patterns.

In our experiments we have deliberately chosen to reproduce classical results, in order to compare the run-time performance of the novel framework to previous implementations of plasticity on SpiNNaker. The examples of BCM, STDP, and voltage-gated STDP learning provide templates for constructing further experiments with rate-based, spike-timing-based, and voltage-dependent learning rules. Our approach can be easily extended to include additional third factors to modulate plasticity, e.g., neuromodulators (Izhikevich, 2007; Potjans et al., 2011), or weight-dependency (Morrison et al., 2008; Nessler et al., 2013), can model homeostatic effects (Bartolozzi et al., 2008), or handle different synaptic delays (Tapson et al., 2013; Wang et al., 2013). It can also combine different models of plasticity in one simulation, a feature which is used in several recent models, where network function arises from the interaction of different synaptic plasticity rules that are specific to particular cell types (Lazar et al., 2009; Savin et al., 2010; Binas et al., 2014; Kleberg et al., 2014). In fact, we have provided a tool that should be general enough to model long-term potentiation rules, but is not restricted only to phenomenological ones. Other biological structures i.e., glial cells are considered to have a fundamental role in plasticity, and can enhance learning capabilities (Min et al., 2012). The plasticity core, by leveraging this functional segregation already present in biology, is a natural candidate to model such structures.

The results presented in this work and the possibilities opened by this approach point to the efficiency and to the generality of the framework introduced: a modular, flexible and scalable tool for the fast and easy exploration of learning models of very different kinds on the parallel SpiNNaker system.

### Conflict of interest statement

The authors declare that the research was conducted in the absence of any commercial or financial relationships that could be construed as a potential conflict of interest.

## Figures and Tables

**Algorithm 1:**
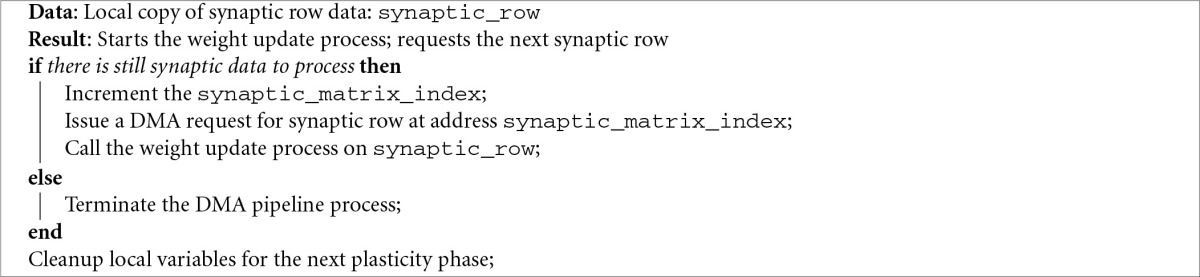
**DMA Pipeline**.

**Algorithm 3:**
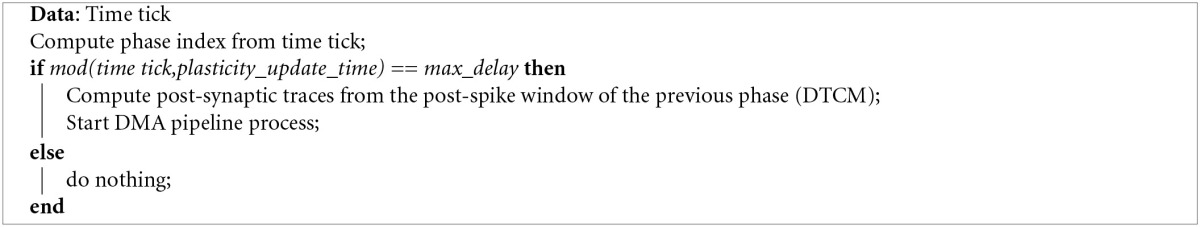
**Timer callback**.

**Algorithm 4:**
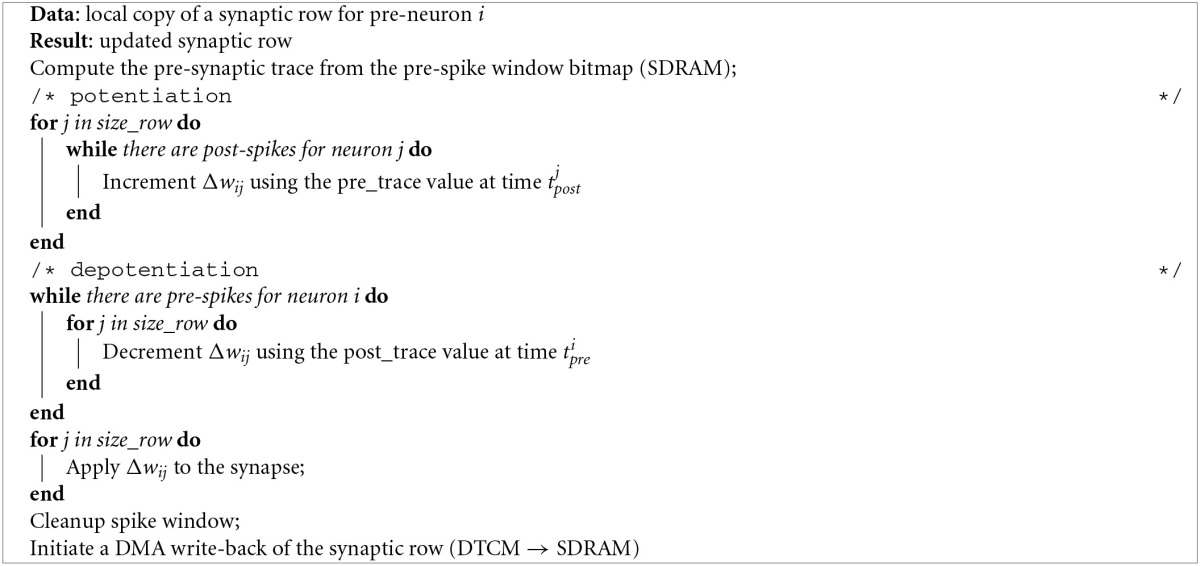
**STDP weight update process**.

**Algorithm 5:**
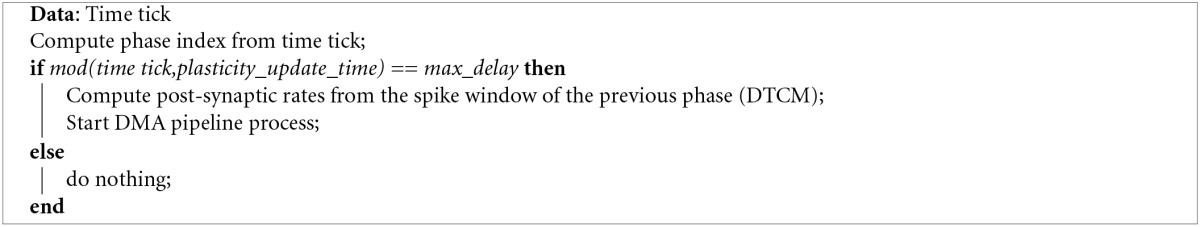
**Timer callback**.

**Algorithm 6:**
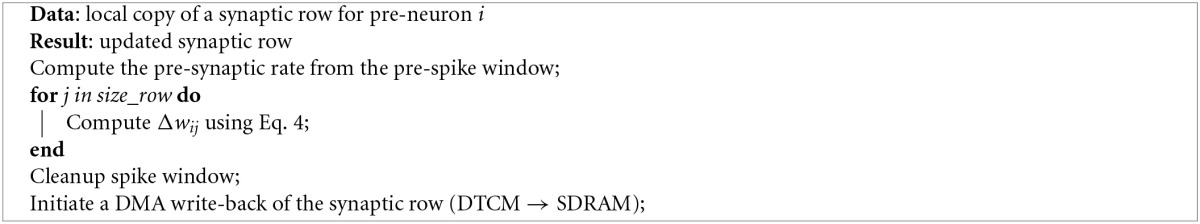
**BCM weight update process**.

**Algorithm 7:**
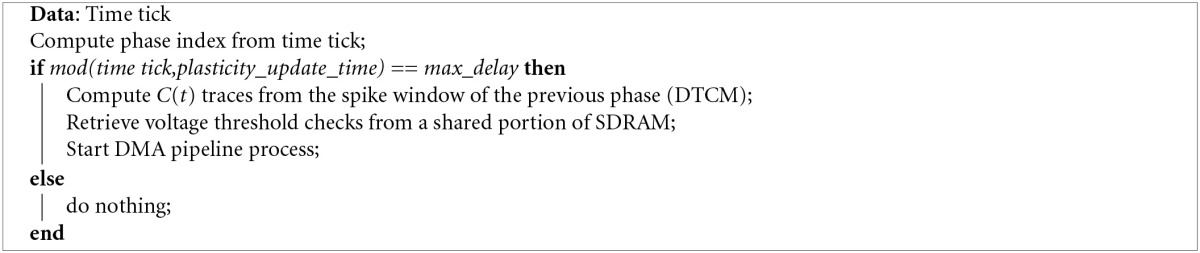
**Timer callback**.

**Algorithm 8:**
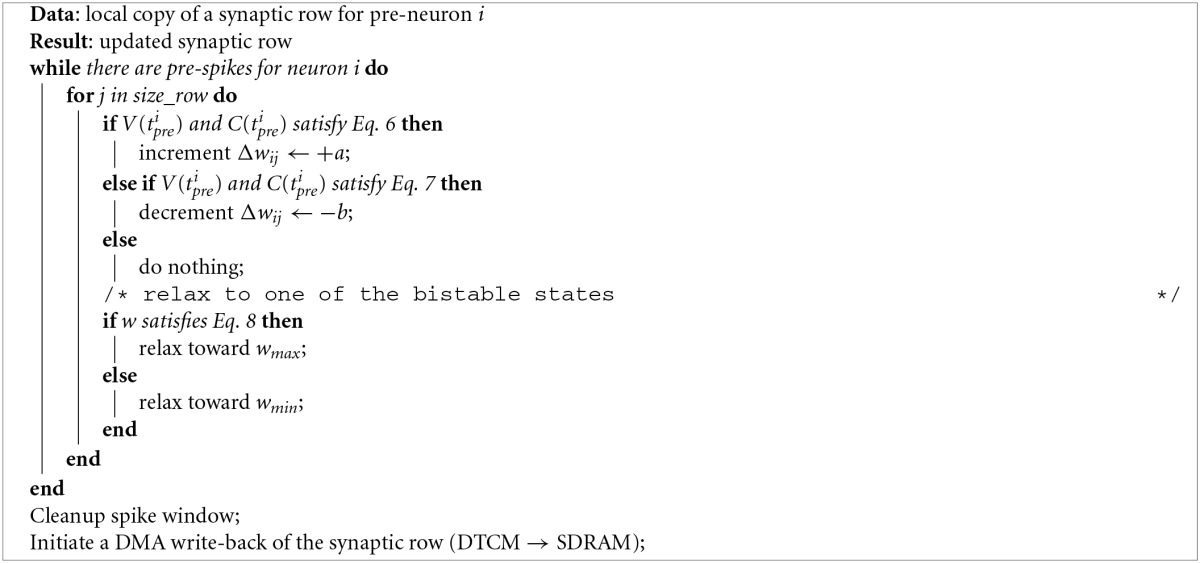
**Voltage-gated STDP weight update process**.

## Footnotes

^1^https://spinnaker.cs.man.ac.uk/tiki-index.php?page=SpiNNaker+Package+(quarantotto)

## Appendix: pseudo code

This appendix presents pseudo code describing the implementation of the common framework and of the rules proposed in this paper. We refer to DTCM for the memory locally available to each ARM core, and to SDRAM as the off-die memory shared by all cores in a chip.

### A common code

The infrastructure for the framework is common for all the learning rules introduced, leaving users free to implement their plasticity rules starting from:

- spike windows containing a compressed form of spike timings for the post-synaptic neurons (stored in DTCM).
- the whole synaptic matrix, indexed by pre-synaptic neuron (stored in SDRAM). Each row contains all the synaptic information needed by the post-synaptic neurons modeled by the neural core for a particular pre-synaptic neuron.
- spike windows containing a compressed form of spike timings for the pre-synaptic neurons (stored at the beginning of each pre-synaptic row in the synaptic matrix).
- additional data needed can be passed through means of shared portions of SDRAM and initialized during the timer callback.

The DMA controller is managing access to SDRAM, relieving the ARM cores of this task and enabling them to perform computation while the memory is fetched or written in parallel. When a core needs to access SDRAM it issues a request to the DMA controller and continues its work. Whenever the requested access is completed a *DMA done interrupt* is generated, triggering a *DMA done callback*.

Synaptic data is indexed by pre-synaptic neuron in SDRAM; each row contains the information about all the core-local post-synaptic neurons each pre-neuron is connected to. Each iteration of the DMA pipeline calls itself on the next portion of the synaptic matrix if there is still data to compute.

The *Packet Received* callback is called whenever a MC packet (containing a post spike) is received, and is used to updated the post-spike window. The mapping software ensures that every spike emitted by the neural core reaches the correspondent plasticity core.

**Algorithm 2:** **Packet Received callback**.

**Data**: Incoming spike (routing key) from the twin neural core
**Result**: Spike window for the current phase
Retrieve the spike window for the post-synaptic neuron (DTCM);
Retrieve the time position for the spike;
Update the spike window;

The *Timer callback* is called every millisecond, and initiates the plasticity process every 128 ms in our implementation. The maximum axonal delay is reintroduced here, to ensure that all the necessary information from the previous phase is present. When initiating the plasticity process rule-specific variables (e.g., traces) can be computed; therefore the timer callback is dependent of each plasticity rule, and described in the next Sections for the three rules implemented.

The *weight update* process is initiated whenever the next row from the synaptic matrix has been retrieved from SDRAM and is locally available to a core. Similarly to the *timer callback*, this function is specific to each plasticity rule implemented and is therefore described in the following Sections.
